# Failure of MMA embolization due to deep and middle temporal artery supply in chronic subdural hematoma: the diagnostic value of 3D-RA

**DOI:** 10.1007/s00701-026-06882-8

**Published:** 2026-04-16

**Authors:** Masaomi Takeuchi, Yoichi Morofuji, Yuki Matsunaga, Chieko Inoue, Eri Shiozaki, Takayuki Matsuo

**Affiliations:** 1 Department of Neurosurgery, Nagasaki University Graduate School of Biomedical Sciences, Nagasaki, Japan; 2 Department of Neurosurgery, Showa Medical University School of Medicine, 1-5-8 Hatanodai, Shinagawa-Ku, 142-8666 Tokyo Japan

**Keywords:** Chronic subdural hematoma, Deep temporal artery embolization, Middle temporal artery embolization, Middle meningeal artery embolization

## Abstract

Chronic subdural hematoma (CSDH) is commonly managed with burr hole evacuation, yet recurrence remains frequent. Middle meningeal artery (MMA) embolization has emerged as a minimally invasive treatment. We describe the case of an 85-year-old man with refractory CSDH who developed multiple recurrences despite repeated surgical evacuation and MMA embolization. Three-dimensional rotational angiography (3D-RA) revealed atypical transosseous feeders from the anterior and middle deep temporal arteries and the middle temporal artery. Targeted embolization using N-butyl cyanoacrylate and coils resulted in durable hematoma resolution without further recurrence. This case highlights the importance of detailed vascular assessment with 3D-RA when MMA embolization fails.

## Introduction

Chronic subdural hematoma (CSDH) is a common condition frequently encountered in daily neurosurgical practice. Burr hole hematoma evacuation has traditionally been the standard treatment; however, recurrence remains a major concern, reported to reach 10–20% [9]. Recently, middle meningeal artery (MMA) embolization has emerged as an alternative or adjunctive therapy to surgical evacuation [3, 15]. This treatment is based on the pathophysiology of CSDH, in which embolization targets the fragile neovascular networks within the hematoma membrane to prevent recurrent bleeding [7]. Here, we report a case of refractory CSDH in which MMA embolization was ineffective; however, successfully managed through embolization of the deep temporal arteries and the middle temporal artery, identified as the dominant sources of blood supply.

## Case report

**Fig. 1:**
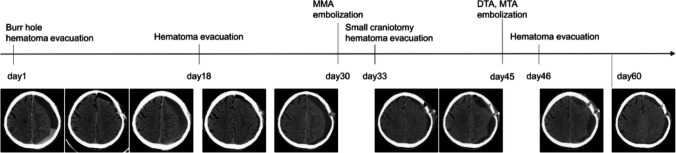
Clinical course of surgical and endovascular interventions with sequential head computed tomography changes. Day 0 indicates the onset. Recurrent left subdural hematoma occurred repeatedly within a short period, even after middle meningeal artery (MMA) embolization. Although some hematoma had remained after the final hematoma evacuation, the hematoma began to decrease two weeks after the deep temporal artery (DTA) and middle temporal artery (MTA) embolization

**Fig. 2:**
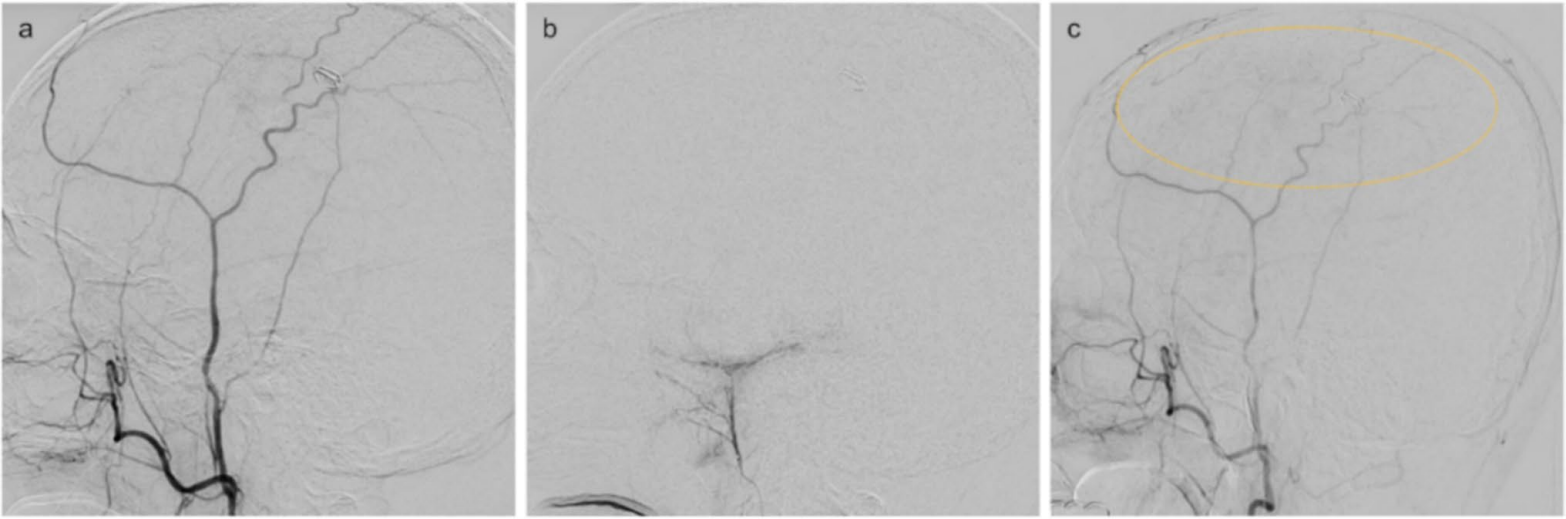
Digital subtraction angiography. The lateral view of the left external carotid artery angiogram (**a**, **c**) and the left middle meningeal artery (MMA) angiogram (**b**) demonstrate the insufficiently developed MMA. Although the extracranial segment of the MMA was embolized using coils, retrospective review suggested additional feeders to the hematoma (dotted circle)

**Fig. 3:**
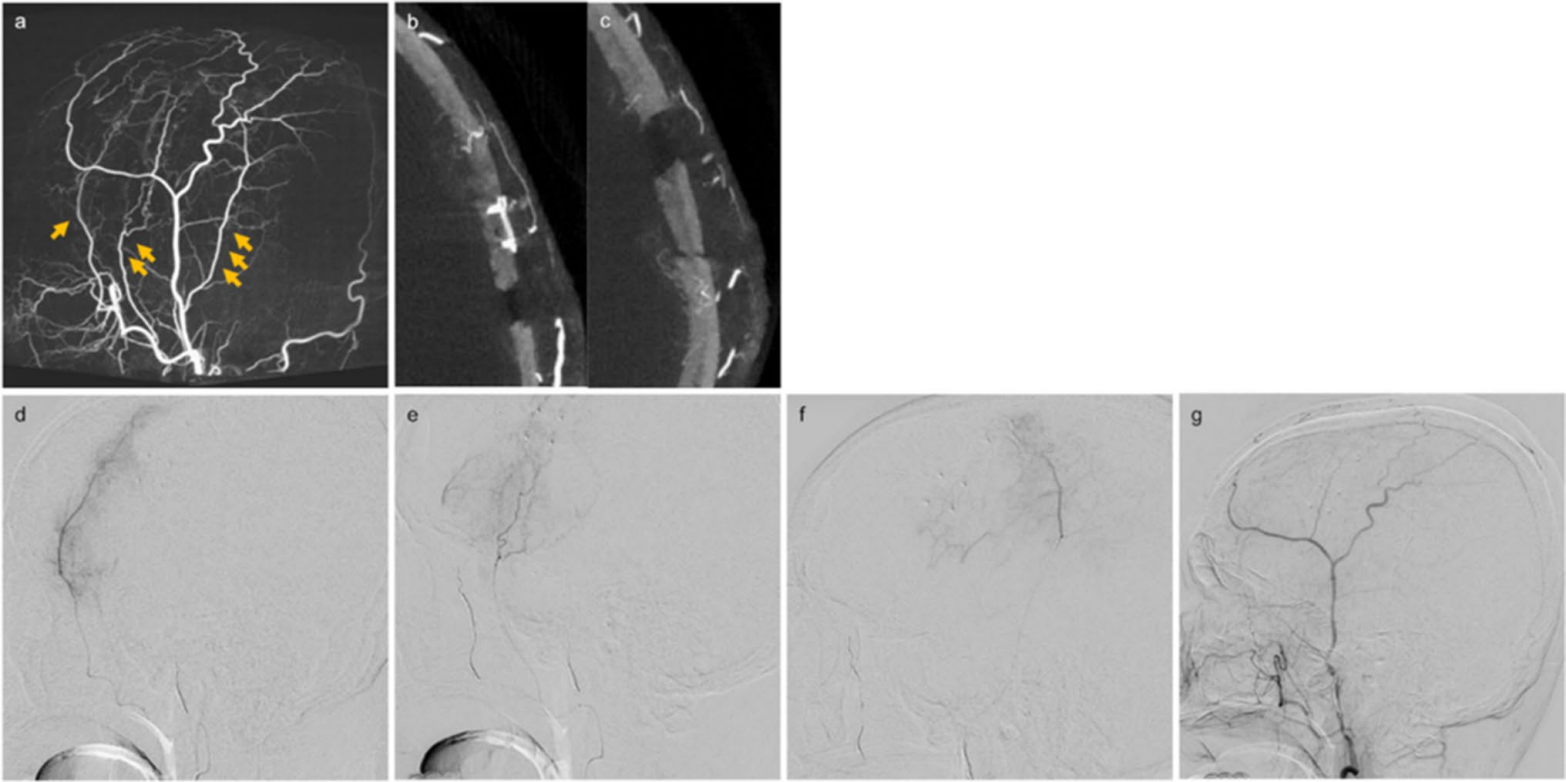
Three-dimensional rotational angiography of the left external carotid artery (**a**: maximum intensity projection [MIP] image) reveals transosseous feeders from the anterior deep temporal artery (ADTA; arrow), middle deep temporal artery (MDTA; double arrows), and middle temporal artery (MTA; triple arrows) to the hematoma outer membrane (**b**, **c**: axial slab MIP image). Lateral views of the left ADTA (**d**), MDTA (**e**), and MTA (**f**) angiograms demonstrate anastomoses among these arteries. Post-embolization lateral view of the external carotid artery angiogram (**g**) shows marked reduction of the abnormal staining

An 85-year-old man with the past medical history of type 2 diabetes, dyslipidemia, hypertension, ischemic heart disease, and endoscopic endonasal sinus surgery for left unilateral invasive fungal sinusitis presented with right hemiplegia followed by rapidly progressive disturbance of consciousness. Computed tomography (CT) revealed a left subdural hematoma (Fig. 1). Burr hole evacuation was performed at another hospital. Two weeks later, right hemiplegia recurred, and a second evacuation for recurrent chronic subdural hematoma was performed. Subsequently, antiplatelet therapy for ischemic heart disease was discontinued. One week later, CT showed hematoma recurrence, and the patient was referred to our department for MMA embolization. Initial digital subtraction angiography (DSA) demonstrated the left MMA was insufficiently developed; however, we performed coil embolization of the extracranial segment with complete obliteration (Fig. 2). Because right hemiplegia appeared, we also performed small craniotomy hematoma evacuation subsequently. Although the symptoms improved temporarily, right hemiplegia and the hematoma recurred. Due to the refractory case with the rapid recurrence, we reviewed the angiographic images obtained during MMA embolization and suspected additional feeders. We performed repeat DSA to consider additional embolization of the responsible vessel. Three-dimensional rotational angiography (3D-RA) revealed transosseous feeders to the hematoma outer membrane arising from the anterior deep temporal artery (ADTA), middle deep temporal artery (MDTA), and the middle temporal artery (MTA), with anastomosis between the MTA and DTAs (Fig. 3a, b, and c). Under local anesthesia, a 6-French sheath (Radifocus introducerⅡH 25 cm; Terumo, Tokyo, Japan) was inserted into the right femoral artery. After systemic heparinization, through a 6-French guiding catheter (Roadmaster 90 cm; Goodman, Aichi, Japan) and a 3.2-French distal access catheter (Guidepost 120 cm; Tokai Medical Products, Aichi, Japan), we advanced a 1.5-French microcatheter (Marathon 165 cm; Medtronic, Minneapolis, Minnesota, USA) over a microguidewire (ASAHI CHIKAI 0.010 inch 200 cm; Asahi Intecc, Aichi, Japan) into the ADTA above the orbital roof to minimize the risk of embolization of the ophthalmic artery. We performed embolization using 12.5% N-butyl cyanoacrylate (NBCA) diluted mixture with lipiodol and coils at the proximal segments after repositioning a 1.7-French microcatheter (Excelsior SL-10 150 cm; Stryker, Kalamazoo, MI, USA) over a microguidewire (ASAHI CHIKAI 0.014 inch 200 cm; Asahi Intecc, Aichi, Japan). Similarly, we performed NBCA embolization via the 1.5-French microcatheter guided to the MDTA and MTA, resulting in marked reduction of the abnormal staining (Fig. 3d, e, f, and g). Subsequently, the fourth time hematoma evacuation was performed using the previous small craniotomy. Although some hematoma had remained presumably due to septation, CT obtained two weeks after the procedure showed that the hematoma had begun to decrease, and no recurrence has been observed since then.

## Discussion

MMA embolization was first reported by Mandai et al. [11] and has recently been reported to be effective as a minimally invasive treatment for recurrent or refractory CSDH [6]. The most common cause of CSDH in the elderly is minor trauma which leads to accumulation of blood mixed with cerebrospinal fluid between the dural border cell layers. This induces an inflammatory response to repair the dural border cell damage forming neomembranes around the hematoma. Angiogenesis within the neomembranes, along with leakage from fragile capillaries, leads to recurrent microbleeds. Some mediators including interleukin-6 and vascular endothelial growth factor (VEGF) are implicated in this process. It has been suggested that the MMA supplies the outer membrane through capillary-like connections that penetrate the dura mater [2, 5]. That is why MMA embolization is considered a pathophysiologically reasonable curative treatment for CSDH. Previous studies have shown that adjunct MMA embolization reduced the recurrence rate from 14.0% to 4.7% compared with craniotomy alone [16]. The benefit is particularly important for elderly patients with a high risk of recurrence or those with comorbidities who are receiving antithrombotic therapy.

**Fig. 4:**
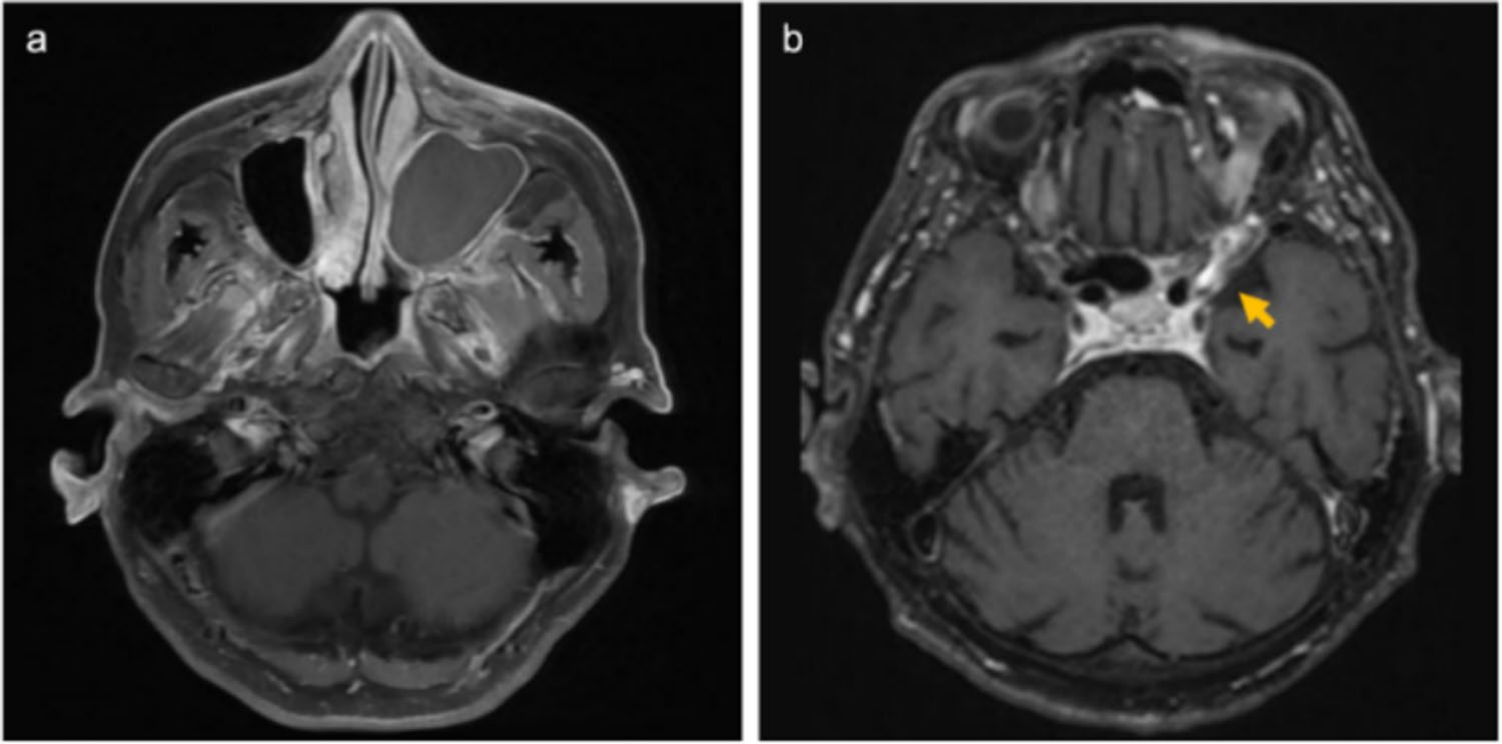
An abnormal signal filling the left maxillary sinus shows hyperintensity on T1-weighted images without appreciable contrast enhancement (**a**). Dural thickening extends from the left cavernous sinus to the middle cranial fossa (**b**, arrow)

In our case, although MMA embolization was performed, the hematoma recurred within a short period of time. Reported mechanisms of CSDH recurrence following MMA embolization include trauma, coagulopathy, organized hematoma, ipsilateral MMA recanalization, and neovascularization from the contralateral MMA [1, 8, 13, 14]. The MMA, which is typically considered the primary feeding artery, was not dominant, whereas the DTA and MTA provided the main supply. Although these vessels are rarely involved, thin-slab maximum intensity projection confirmed transosseous feeders to the hematoma outer membrane. To the best of our knowledge, no previous reports have demonstrated anastomoses between the DTA and MMA without passing through the orbit. It is possible that MMA degeneration occurred as a result of invasive fungal sinusitis [12], with inflammation extending from the maxillary sinus to the dura mater of the middle cranial fossa (Fig. 4). Consequently, the DTA and MTA may have formed transosseous anastomoses with peripheral branches of the MMA, becoming the dominant supply to the hematoma, possibly facilitated by VEGF-mediated vascular proliferation. Despite the presence of these feeders during the initial treatment, they were not recognized because of their atypical appearance. A retrospective review following recurrence, together with subsequent 3D-RA, enabled definitive identification.

In addition, the choice of embolic agents may influence treatment outcomes. Liquid embolic agents allow deeper penetration into distal neovascular networks, whereas proximal coil embolization is often selected to reduce the risk of non-target embolization and has been reported as a safe and effective alternative [4]. In the present case, proximal coil embolization was initially selected because the middle meningeal artery was insufficiently developed. However, treatment failure was more likely attributable to unrecognized transosseous feeders from the DTA and MTA than to the choice of embolic agents. These findings emphasize that accurate identification of all feeding arteries is essential for determining the optimal embolization strategy.

To our knowledge, this is the first report of successful embolization of both the DTA and MTA for refractory CSDH, although a single prior case of DTA-only embolization has been reported [10]. This case highlights that CSDH can involve atypical and diverse vascular networks, and that detailed vascular assessment, particularly with 3D-RA, may be crucial when MMAE fails or when the vascular supply appears atypical. Although the outcome was favorable, larger studies are required to evaluate the efficacy and safety of targeting alternative feeding vessels beyond the MMA.

## Conclusion

In case of refractory CSDH without typical blood supply from the MMA, alternative vascular sources could be involved. Detailed vascular structures assessment using techniques such as 3D-RA is crucial for endovascular treatment of refractory CSDH.

## Data Availability

No datasets were generated or analysed during the current study.
